# Are 6 more accurate than 4? The influence of different modes of delivery on postpartum depression and PTSD

**DOI:** 10.1186/s12884-024-06267-8

**Published:** 2024-02-08

**Authors:** Franziska Marie Lea Beck-Hiestermann, Lisa Kathrin Hartung, Nadine Richert, Sandra Miethe, Silke Wiegand-Grefe

**Affiliations:** 1 Department of Psychology, Medical School Hamburg, Hamburg, Germany; 2 Department of Psychosomatic Medicine and Psychotherapy, Psychologische Hochschule Berlin, Berlin, Germany; 3 Clinic of Psychiatry, Socialpsychiatry and Psychotherapy, Hannover Medical School, Hannover, Germany; 4 Institute for Clinical Psychology and Psychotherapy, Medical School Hamburg, Hamburg, Germany; 5 Department of Child and Adolescent Psychiatry and Psychotherapy, University Medical Center Hamburg-Eppendorf, Hamburg, Germany

**Keywords:** Postpartum depression (PPD), Post-traumatic stress disorder (PTSD), Caesarean section (CS), Vaginal delivery, Assisted vaginal delivery (AVD), Birth, Postpartum psychological disorders

## Abstract

**Background:**

Empirical evidence shows that 4.6–6.3% of all women develop a post-traumatic stress disorder (PTSD) and approximately 10–15% postpartum depression (PPD) following childbirth. This study explores the relationship between delivery mode and the occurrence of PTSD and PPD, specifically examining four distinct caesarean section (CS) modes: primary on maternal request (Grade 4), medically indicated primary (Grade 3), secondary CS from relative indication (Grade 2) and emergency secondary CS (Grade 1), compared to vaginal and assisted vaginal delivery (AVD). The research aims to understand how these six subcategories of delivery modes impact PPD and PTSD levels. Common predictors, including the need for psychological treatment before childbirth, fear of childbirth, planning of pregnancy, induction of labor, birth debriefing, and lack of social support after childbirth, will be analyzed to determine their association with postpartum mental health outcomes.

**Methods:**

The study was planned and carried out by a research team of the psychology department at the Medical School Hamburg, Germany. Within an online-study (cross-sectional design) *N* = 1223 German speaking women with a baby who did not die before, during or after birth were surveyed once between four weeks and twelve months postpartum via an anonymous online questionnaire on demographic and gynecological data, delivery mode, PTSD (PCL-5) and PPD (EPDS).

**Results:**

For both psychiatric disorders, ANOVA revealed significant differences between delivery mode and PPD and PTSD. With weak effects for PPD and medium to strong effects for PTSD. Post-hoc tests showed increased levels of PPD for two CS types (Grade 1, Grade 3) compared to vaginal delivery. For PTSD, secondary CS from relative indication (Grade 2), emergency secondary CS (Grade 1) and assisted vaginal delivery (AVD) were associated with elevated levels of PTSD. Regression analysis revealed delivery mode as a significant predictor of EPDS- (medium effect size) and PCL-5-Score (medium to high effect size).

**Limitation:**

Delivery was considered as the potential traumatic event, and any previous traumas were not documented. Additionally, the categorization of delivery modes relied on subjective reports rather than medical confirmation.

**Conclusion:**

The study highlights the influence of delivery mode on the mental health of postpartum mothers: different modes influence postpartum disorders in various ways. However, the definition of delivery mode was only stated subjectively and not medically confirmed. Further research should investigate which aspects of the different delivery modes affect maternal mental health and explore how the perception of childbirth may be influenced by specific delivery experiences.

## Introduction

Although the act of giving birth has a predominantly positive connotation in Western society, it still poses a risk for the development of a variety of mental disorders, such as postpartum depression (PPD) or post-traumatic stress disorder (PTSD) after a traumatic delivery (e.g. [1, 2]). .

Postpartum psychiatric disorders are relatively common, with 10–15% of mothers experiencing PPD [3], and some studies reporting rates up to 19.8% [4]. Additionally, 4.6–6.3% of mothers suffer from PTSD related to childbirth, with higher rates in at-risk groups (e.g. maternal history of trauma, peripartum complications) [5, 6]. These disorders not only affect mothers’ quality of life but also extend to relationship problems with partners [7] and impact on infant emotional regulation and development [8–10].

Extensive research has been conducted to investigate the prenatal and perinatal risk factors for various postpartum mental disorders [11–17]. In particular for PPD social risk factors (i.e., age, low socioeconomic status, planning of pregnancy), psychological risk factors (i.e., history of mental disorders, fear of childbirth, lack of social support after birth), and biological risk factors (i.e., chronic or medical illnesses) are considered well established [17].

There is less research on risk factors for the development of PTSD following childbirth, but psychological factors (history of mental disorders, fear of childbirth), medical factors (induction of labor, birth debriefing) and social factors (age, lack of social support after childbirth) are also frequently observed [18, 19]. Part of the research addresses the subjective birth experience as a possible mediating factor [19, 20].

While various risk factors contribute to maternal mental health, some studies have focused on the effect of delivery modes and reviewed its influence on maternal mental health [21]. Caesarean sections (CS) were associated with a more negative view of childbirth, the self, and the infant. Moreover, feelings of failure, self-blame and reduced self-esteem have commonly been reported after CS [21]. Such results show that CS may have adverse effects on maternal mental health. However, as CS were only categorized into two different types (CS on maternal request and emergency CS) it remains unclear whether further, more specific categorizations could provide additional insights.

A meta-analysis has shown that CS may increase the risk of PPD [22]. Women who undergo CS, whether planned or unplanned, tend to exhibit higher levels of somatization, depression, and anxiety symptoms compared to those with vaginal deliveries [5], indicating that CS might lead to greater general distress and poorer mental health, including PPD. Additionally, a systematic review and meta-analysis found higher depression levels associated with CS, regardless of categorization, compared to vaginal deliveries [23, 24].

The increased risk of PPD following CS compared to vaginal delivery is not fully understood. While CS disrupts the normal labor process and hormonal environment, potentially affecting maternal mental health [25], assisted vaginal deliveries (AVD) also impact mental health [5], suggesting hormonal changes in CS may not be the leading cause. Lower maternal satisfaction after a CS could play an important role when assessing the impact on maternal mental health. As mentioned above, women who had a CS often battle with feelings of failure or reduced self-esteem [21], possibly because they feel like they were not able to give birth “the right way”. This could lead to repetitive negative thinking, a strong predictor of depressive symptoms [26]. However, some research has not reached the same conclusions. A review found no evidence for a link between CS and PPD [27], and no differences in PPD levels between vaginal deliveries, CS on maternal request or emergency CS were found [28]. Another research group also concluded that a CS does not pose a greater risk for PPD in the medium to long-term after delivery [29]. However, comparability of existing studies is low due to methodological differences. While some only compare CS and vaginal deliveries [23], others differentiate between maternal request and emergency CS [28]. Not all studies distinguish between normal vaginal delivery and AVD, although AVD is associated with increased risk for traumatic potential [30, 31]. These methodological discrepancies may explain the inconclusiveness of existing evidence emphasizing the need for further studies that more precisely distinguish between specific types of CS [22].

The British Royal College of Obstetricians and Gynecologists (RCOG) recommends to categorize CS in a more comprehensive manner to account for their differences based on the urgency with which a CS is performed. This results in six different categories; (1) vaginal delivery, (2) AVD (involving the use of vacuum or forceps to guide the infant out of the birth canal), (3) CS on maternal request (Grade 4), (4) medically indicated CS (Grade 3), (5) secondary CS (Grade 2) and (6) emergency CS (Grade 1) [32]. A CS on maternal request is pre-planned and performed before labor begins at the mother’s request, while a medically indicated CS is also pre-planned but performed for medical reasons (e.g., transverse fetal presentation) without maternal or fetal compromise. A secondary CS is conducted after labor onset due to maternal or fetal compromise but without immediate life-threatening risks (e.g., cord prolapse, fetal distress, dystocia). Emergency CS are performed in response to immediate threats to the mother or fetus during labor, requiring quick decisions and often involving higher stress and potential complications. In contrast, CS on maternal request are pre-scheduled, allowing for better preparation and management, reducing unexpected complications and providing a more controlled surgical environment.

The differentiation proposed by the RCOG distinguishes more accurately the causes for different modes of delivery and thus was used in this study to examine the association between delivery mode and mothers’ psychopathology and other psychological outcomes postpartum.

The following questions will be investigated:

1. What proportion of women meet the cut-off criterion of the questionnaires used to measure PPD (EPDS) and PTSD (PCL-5)?
2. Are there mean differences between the six birth modes in terms of the level of PPD or PTSD scores?
3. Do the six delivery modes predict levels of PPD and PTSD beyond the common risk factors?

## Methods

### Sample

This study was planned and carried out by a research team at the psychology department of the Medical School Hamburg, Germany. Utilizing an online cross-sectional design, a total of *N* = 1223 mothers who were between four weeks and 12 months post-delivery participated in the study. The participants were recruited between 11/2018 and 03/2019 using social media (postings the study-link in birth related groups on Facebook and hashtags targeting new mothers on Instagram). No financial benefit was offered, and the participation was voluntary. The study is carried out according to the Good Clinical Practice (GCP) guidelines, the Declaration of Helsinki. In the initial phase of the survey, the participants received detailed written explanation of the study and were informed that some of the questions might relate to unpleasant or even traumatic experiences, which might trigger unwanted memories and emotions. Participants could electronically give their consent, were informed about their right to withdraw at any time and were encouraged to seek professional help if needed (informed consent included a list of mental health services like the German National Crisis Line). The study included all German-speaking mothers who were at least 18 years old. Women who lost their child during or shortly after birth were excluded so that possible symptoms are not due to a reaction of grief.

### Measures

*Demographic and gynecological data*: included general demographic data such as age, residential environment, number of children, occupational situation, educational and family status. In addition, gynecological data were collected. These included the date, place and type of delivery and the desired delivery, the planning of pregnancy, high-risk or twin pregnancy, miscarriages, current pregnancy, and induction of labor. Also, the need for psychological treatment before childbirth and post-birth debriefing were assessed with a yes/no question. Additionally, this study utilized specially created items, each with a five-level Likert scale, to examine fear of childbirth (never – seldom – sometimes -often -always) and the extent of social support after childbirth (not at all – not very – sometimes – very – absolutely).

*Mode of Delivery*: The mothers had to indicate the delivery mode via self-report. The distinction is made between vaginal, assisted vaginal delivery and four CS modes: primary CS on maternal request (Grade 4), primary CS with medical indication (Grade 3), secondary CS without emergency character (Grade 2) and emergency secondary CS (Grade 1).

*Depression*: was recorded with the Edinburg Postnatal Depression Scale [33]. The German version was used [34], which has a Cronbach’s Alpha of α = 0.81. This is the only validated German instrument for recording symptoms of PPD. The questionnaire contains ten items, each are asked for using the Likert scale from 0 (not at all) to 3 (yes, very often). Thus, the range of the measured value extends from 0 (no symptoms) to 30 (very severe symptoms). The recommended cut-off indicating the need of further diagnostic assessment is ≥ 10 [33, 34], with a sensitivity for PPD of 0.84 and a specificity of 0.84.

*PTSD*: was assessed using the Post-traumatic Stress Disorder Checklist (PCL-5) [35], German version [36], a newer diagnostic tool that has been adapted to the changed criteria for diagnosing PTSD according to DSM-5. Each item from the 20-item scale is assessed using a five-level Likert scale from 0 (not at all) to 4 (very strong). Higher values represent a stronger expression of the symptom, thus a maximum value of 80 is possible. Above a cut-off value of ≥ 33, further assessment is recommended [35, 36], e.g., in the form of a structured clinical interview. At a cut-off value of ≥ 33 the PCL-5 has a sensitivity of 0.86 and a specificity of 0.68 to identify PTSD.

### Statistical analyses

All analyses were performed with IBM SPSS 25.

ANOVAs were used to compare the six delivery modes regarding differences in PCL-5- and EPDS-Scores. One ANOVA was performed for the dependent variable EPDS-Score and one ANOVA for the dependent variable PCL-5-Score.

Block-wise, multiple regressions were conducted for EPDS- and PCL-5-Score. After controlling for age and educational level (dummy coded, high – medium – low, with high as reference category) common predictors were entered in the regression model (all coded: yes versus no). Those were for EPDS-Score: the need for psychological treatment before childbirth, fear of childbirth, planning of pregnancy, lack of social support after childbirth and for PCL-5-Score: the need for psychological treatment before childbirth, fear of childbirth, induction of labor, birth debriefing and lack of social support after childbirth. In a last step the delivery mode was entered in both models (dummy coded, AVD – Grade 1 – Grade 2 – Grade 3 – Grade 4, with vaginal as reference category).

Statistical significance was evaluated two-sided at the 5% level.

## Results

### Sample

A total of *n* = 1223 mothers took part in the survey, participants were mothers with a median age of 28.89 years (SD = 4.09), 66.6% were married, 39.1% had a high educational level, and the average number of children was 1.47 years (SD = 0.67). Table 1 provides an overview of the demographic and obstetric data. Additionally, wherever feasible, relevant comparisons were drawn with statistics from the German population.

### Modes of delivery

The six modes of delivery are represented as follows: 57.5% (*n* = 703) delivered vaginally, *n* = 145 (11.9%) had an assisted vaginal delivery. The CS types were divided into *n* = 26 women (2.1%) who had Grade 4, *n* = 119 women (9.7%) who had Grade 3, *n* = 131 (10.7%) who had Grade 2 and *n* = 99 women (8.1%) who delivered per Grade 1. In summary, this corresponds to a CS rate of 30.6%.

**Table 1:** Sample characteristics

	sample				
	n		%	M (SD)	German population*
total		1223			
age				28.89 (4.09)	30.0
relationship status					
married		815	66.6		51.2%
partner or engaged		371	30.3		
no partner, widowed, divorced		37	3.1		
educational level					
high		478	39.1		32%
medium		426	34.8		52%
low		319	26.1		16%
number of children				1.42 (0.67)	1.46
high-risk pregnancy		274	22.4		34.9%
twin pregnancies		74	6.1		1.7%
fetal presentation at childbirth		848			
cephalic		739	87.1		92%
breech		90	10.6		5%
transversal		1	0.1		0.3%
unsure		18	2.1		/
delivery mode**:					
vaginal		703	57.5		63.2%
AVD		145	11.9		6.1%
Grade 4		26	2.1		
Grade 3		119	9.7		
Grade 2		131	10.7		
Grade 1		99	8.1		
CS total		375	30.6		30.9%***

* Data originate from the German Federal Statistical Office (2021), they are presented as no. or % unless otherwise indicated

** delivery mode: CS = caesarean section, AVD = assisted vaginal delivery (involving the use of vacuum or forceps to guide the infant out of the birth canal), Grade 4 = CS on maternal request, Grade 3 = medically indicated CS, Grade 2 = secondary CS, Grade 1 = emergency CS

*** According to the Federal Statistical Office, the types of CS (Grade 1 to 3) in Germany are not surveyed individually; only CS on maternal request are recorded individually

Of all participants, *n* = 1.082 (88.5%) wanted a vaginal delivery. *N* = 49 (4%) expressed a preference for CS as their chosen delivery mode, while *n* = 92 (7.5%) had no fixed preference.

On average, the delivery took place in the 39th week of pregnancy (M = 39.36, SD = 2.27, minimum 25, maximum 43). 66.6% of the women (*n* = 814) were first-time mothers. 22.4% (*n* = 274) meet the criteria for high-risk pregnancy.

### Postpartum depression

The mean value of the EPDS sum score for the entire sample is M = 7.58 (SD = 5.57), the maximum total score is 30. Women with a Grade 4 CS achieve the lowest value (M = 6.31; SD = 5.33). This is followed by the group with an AVD (M = 7.08, SD = 5.51), followed by women with a Grade 2 (M = 7.18; SD = 5.31). Women with vaginal delivery scored M = 7.35 (SD = 5.49), followed by women with Grade 3 CS (M = 7.82; SD = 5.36). On average, women with a Grade 1 (M = 10.52; SD = 6.09) achieved the highest score.

**Table 2:** Scores for postpartum depression and post-traumatic stress symptoms for the different types of delivery

	***N*** total sample	EPDS cut-off ≥ 10 fullfilled		total sample	***n***	PCL-5 cut-off ≥ 33 fullfilled	total sample
		*n*	%	M (SD)		%	M (SD)
total	1223	395	32.30	7.58 (*5.57*)	97	7.93	11.84 (12.10)
vaginal	703	207	29.45	7.35 (5.49)	30	4.27	9.53 (10.14)
AVD	145	43	29.66	7.08 (5.51)	19	13.10	14.65 (14.42)
Grade 4	26	8	30.77	6.31 (5.33)	0	0.00	8.54 (9.07)
Grade 3	131	43	32.82	7.82 (5.36)	6	5.04	11.71 (11.18)
Grade 2	119	40	33.61	7.18 (5.31)	13	9.92	13.08 (12.22)
Grade 1	99	54	54.55	10.53 (6.09)	29	29.29	23.59 (14.80)

AVD = assisted vaginal delivery (involving the use of vacuum or forceps to guide the infant out of the birth canal), Grade 4 = caesarean section (CS) on maternal request, Grade 3 = medically indicated CS, Grade 2 = secondary CS, Grade 1 = emergency CS

### PTSD

The PCL-5 total mean value of all respondents is M = 11.84 (SD = 12.10), with a total value of 80 representing the maximum value. Table 2 gives an overview of the mean values per delivery mode. The lowest mean value for the Grade 4 group is M = 8.54 (SD = 9.07). For women with vaginal delivery, this is M = 9.53 (SD = 10.14). Participants with Grade 3 have a value of M = 11.71 (SD = 11.18), women with Grade 2 M = 13.08 (SD = 12.22), followed by the group with an AVD with M = 14.65 (SD = 14.42). The highest average value was achieved by women with Grade 1 CS (M = 23.59, SD = 14.80).

### Analysis of variance

#### Delivery modes and postpartum depression

The homogeneity of variance was tested by means of a Levene’s test, according to which an equality of the variances could be assumed (*p* = .327).

A one-way ANOVA was performed. There was a statistically significant difference in EPDS scores for the different modes of delivery, *F*(5, 1217) = 6,552, *p* < .001, with a small effect (ƞp² = 0.026).

#### Delivery modes and postpartum PTSD

First, the homogeneity of variance was tested by means of a Levene’s test. According to this test, no homogeneity of the variances can be assumed (*p* < .001).

Subsequently, a one-way ANOVA was carried out here as well. The severity of PTSD (measured by PCL-5) differed statistically significant for the different delivery modes, *F*(5, 1217) = 28.99, *p* < .001, ƞp² = 0.11. There was a medium to strong effect of ƞp² = 0.11.

### Regression analysis

#### EPDS

In order to explore the influence of educational level, age, the need for psychological treatment before childbirth, fear of childbirth, planning of pregnancy, lack of social support after childbirth and delivery mode on EPDS-Score hierarchical multiple regression analyses were conducted.

In the first step potential control variables age (β = − 0.064, *p* < .05) and educational level (medium: β = 0.132, *p* < .001 and low: β = 0.105, *p* < .001) were entered in the regression model to assess their influence on EPDS-Score. This first step explained a significant amount of variance R² adjusted = 0.031, (*p* < .001).

Adding the need for psychological treatment before childbirth (β = − 0.130, *p* < .001), fear of childbirth (β = 0.208, *p* < .001), planning of pregnancy (β = 0.058, *p* < .05) and lack of social support after childbirth (β = 0.135, *p* < .001) in a second step, the explained variance increased significantly (*p* < .001) to R² adjusted = 0.122 (meaning 12.2% more variance is explained).

In a third step, the delivery mode variable was added (AVD: β = − 0.013, *p* = .638, Grade 4: β = − 0.020, *p* = .466, Grade 3: β = 0.003, *p* = .904, Grade 2: β = − 0.017, *p* = .538, Grade 1: β = 0.121, *p* < .001). Adjusted R² in the final model for EPDS-Score was R² = 0.135 (meaning 13.5% of variance is explained), thus also declaring a significant amount of variance (*p* < .001).

The R² for the overall model was R² =0.143 (adjusted R² = 0.135), indicative for a mean goodness-of-fit [37]. Accordingly, the nine predictors significantly predicted depression (as measured by the EPDS) and were able to explain 13.5% of the variance.

Nevertheless, the change in R² from step 2 to step 3 amounts to only ΔR² = 0.016, which provides a small amount of variance resolution despite significance (*p* < .05). See Table 3 for a summary of the hierarchical multiple regression analyses.

**Table 3:** EPDS- hierarchical multiple regression analyses

model	predictors	EPDS		
		β	t	R²adj
**step 1**				.031**
	age	− .064*	-2.184	
	educational level			
	medium	.132**	4.528	
	low	.105**	3.632	
**step 2**				.122**
	age	− .048	-1.684	
	educational level			
	medium	.109**	3.927	
	low	.068*	2.443	
	psychological treatment before childbirth	− .130**	-4.783	
	fear of childbirth	.204**	7.648	
	planning of pregnancy	.058*	2.084	
	lack of social support after childbirth	.130**	-4.783	
**step 3**				.135**
	age	− .052	-1.815	
	educational level			
	medium	.103**	3.708	
	low	.065*	2.346	
	psychological treatment before childbirth	− .127**	-4.675	
	fear of childbirth	.198**	7.290	
	planning of pregnancy	.057*	2.045	
	lack of social support after childbirth	.132**	4.912	
	delivery mode***			
	AVD	− .013	-0.471	
	Grade 4	− .020	-0.730	
	Grade 3	.003	0.121	
	Grade 2	− .017	-0.616	
	Grade 1	.121**	4.409	
**ΔR² from step 1 to step 2**			.093**	
**ΔR² from step 2 to step 3**			.016*	

The explained variances are reported as adjusted R²

**p < .01, *p < .05: significance of increase in explained variance and significance of beta weights

***delivery mode: AVD = assisted vaginal delivery (involving the use of vacuum or forceps to guide the infant out of the birth canal), Grade 4 = caesarean section (CS) on maternal request, Grade 3 = medically indicated CS, Grade 2 = secondary CS, Grade 1 = emergency CS

#### PCL-5

To investigate the influence of the variables educational level, age, the need for psychological treatment before childbirth, fear of childbirth, lack of social support after childbirth, delivery mode, induction of labor and birth debriefing also on the PCL-5-Score, a stepwise hierarchical multiple regression analysis was performed.

In the first step potentially confounding variables age (β = − 0.131, *p* < .001) and educational level (medium: β = 0.113, *p* < .001 and low: β = 0.087, *p* < .05) were entered in the regression model. This step explained a significant amount of variance R² adjusted = 0.041, (*p* < .001).

With the addition of the predictors in a second step, the explained variance increased significantly to R² adjusted = 0.119 (meaning 11.9% of variance is explained), (*p* < .001). The beta weights of the predictors were as follows: the need for psychological treatment before childbirth (β = − 0.090, *p* = .001), fear of childbirth (β = 0.166, *p* < .001), induction of labor (β = 0.066, *p* = .013), birth debriefing (β = 0.067, *p* = .015) and lack of social support after childbirth (β = 0.109, *p* < .001).

In a final step, the delivery mode variable was added (AVD: β = 0.132, *p* < .001, Grade 4: β = 0.011, *p* = .679, Grade 3: β = 0.060, *p* = .027, Grade 2: β = 0.081, *p* < .05, Grade 1: β = 0.293, *p* < .001). This addition significantly improved the explained variance to R² adjusted = 0.204, (*p* < .001).

The R² for the overall model was R² =0.213 (adjusted R² = 0.204), indicative for a mean to high goodness-of-fit according [37]. Accordingly, the nine predictors significantly predicted PTSD (as measured by the PCL-5) and were able to explain 20.4% of the variance. The change in R² from step 2 to step 3 amounts to ΔR² = 0.088, suggesting that the delivery mode variable is a meaningful predictor for the PCL-5-Score.

See Table 4 for a summary of the hierarchical multiple regression analyses.

**Table 4:** PCL-5 - hierarchical multiple regression analyses

model	predictors	PCL-5			
		β		t	R²adj
**step 1**					.041**
	age	− .131**		-4.511	
	educational level				
	medium	.113**		3.895	
	low	.087*		3.042	
**step 2**					.125**
	age	− .121**		-4.331	
	educational level				
	medium	.088*		3.136	
	low	.057*		2.067	
	psychological treatment before childbirth	− .085*		-3.140	
	fear of childbirth	.192**		7.046	
	induction of labor	.086*		3.204	
	birth debriefing	0.55*		1.918	
	lack of social support after childbirth	.115**		4.026	
**step 3**					.204**
	age	− .135**		-5.019	
	educational level				
	medium	.058*		2.157	
	low	.049		1.860	
	psychological treatment before childbirth	− .090*		-3.484	
	fear of childbirth	.166**		6.362	
	induction of labor	.066*		2.498	
	birth debriefing	.067*		2.428	
	lack of social support after childbirth	.109**		3.976	
	delivery mode***				
	AVD	.132**		4.991	
	Grade 4	.011		0.414	
	Grade 3	.060*		2.212	
	Grade 2	.081*		3.043	
	Grade 1	.293**		11.087	
**ΔR² from step 1 to step 2**			.081**		
**ΔR² from step 2 to step 3**			.088**		

The explained variances are reported as adjusted R²

**p < .01, *p < .05: significance of increase in explained variance and significance of beta weights

***delivery mode: AVD = assisted vaginal delivery (involving the use of vacuum or forceps to guide the infant out of the birth canal), Grade 4 = caesarean section (CS) on maternal request, Grade 3 = medically indicated CS, Grade 2 = secondary CS, Grade 1 = emergency CS

## Discussion

This study investigated the relationship between delivery modes and postpartum psychiatric symptoms associated with PPD and PTSD.

It was tested as to whether the delivery mode (subdivided into six categories) affects the level of PPD and PTSD and the impact as a risk factor beyond the common ones. Descriptive analyses showed that the depression score (measured by EPDS) was significantly higher than reported in the literature. Within the sample, 32.30% achieved a cut-off score ≥ 10, which contrasts with the number of 10–15% literature-based prevalence [38]. However, it should be noted that in other countries a higher cut-off value of 12 or 13 is suggested for the EPDS, potentially impacting the interpretation of our results. Analysis of variance showed a significant difference between delivery modes with small effect size. However, delivery modes need to be differentiated. Post hoc tests showed that women with Grade 1 (emergency CS) had the highest depression scores (54.55% above cut-off), but Grade 2 (secondary CS) and Grade 3 (CS for medical reasons) were also associated with higher depressive scores (Grade 3: 33.61%, Grade 4: 32.82% above cut-off). Grade 1 (emergency CS) arises from a critical situation for both the mother and child, necessitating an immediate shift from vaginal birth. There is no alternative option for the mother, which can lead to feelings of being abandoned. Whereas in Grade 2 there is a soft indication and the secondary CS is one option among others. The mother can be involved in the decision, although the decision-making process can be overwhelming in the extreme situation of childbirth. At Grade 3, the indication for a CS is made before the onset of labor. The mother has more time to mentally prepare for the CS. Nevertheless, not being able to give birth in the desired mode could lead to higher rates of depressive symptoms.

88.5% of the subjects expressed a preference for vaginal delivery. Women with desired mode of delivery were likely to experience themselves as self-efficacious and therefore had a lower depression score. If women lack this experience of giving birth in a “natural” (and often socially idealized) way, they may blame themselves for not making it and feel feelings of failure or guilt [39], which in turn is associated with higher depression scores. This is supported by the findings of a recent German study [40], indicating that women who do not undergo a natural childbirth are more prone to experiencing feelings of guilt and higher depressive symptomatology. Following on from this it seems explainable, that although AVD is associated with worse delivery experiences [41], women had no elevated depression levels. Because AVD it is still a vaginal delivery and could lead to a sense of pride and therefore be protective. It is also imaginable that these effects could be due to more difficult attachment, which occurs more frequently after CS [42, 43].

Another possible explanation for the elevated depression scores in Grade 1 is that the decision to perform an emergency CS is often made under time pressure, possibly without the woman’s explicit consent. In addition to lacking self-efficacy expectations, this delivery mode is often associated with fear, especially fear for the life of the child [44]. And this, in turn, is associated with a higher likelihood of developing PPD. The overall small effect of the analysis of variance (ƞp² = 0.026) allows for the interpretation that there are other factors influencing the development of PPD. This is also reflected in the regression analysis, in which delivery mode was a significant predictor, but the variance explained by it was small.

Regarding PTSD (measured with PCL-5), 7.93% of all women met the cut-off of ≥ 33. This rate is also significantly higher than the prevalence reported in the literature, which is 2–6% on average [5, 45]. Nevertheless, it should be noted that the PCL-5-Scores do not equate to a confirmed diagnosis. About one third of the subjects (29.29%) with Grade 1 (emergency CS) were above the cut-off. This could be explained by the life-threatening nature of emergency CS. The subjectively perceived threat, the danger of physical integrity for mother and child as well as the actual injuries that occur in Grade 1 CS correspond to the trauma-criterion. Women with secondary CS and thus soft indication (Grade 2), on the other hand, meet the cut-off for PTSD more frequently than reported prevalences (9.92%), but by far not as frequently as women with emergency CS (Grade 1). Hence, the latter appears to possess a distinct characteristic in terms of trauma genesis. It stands out from all other delivery modes due to its emergency nature and the acute risk it poses to both mother and child.

This confirms by findings, which revealed the absence of perceived safety during childbirth as a significant predictor of the development of PTSD [46]. Childbirth inherently represents an exceptionally intense situation, involving both physical pain and psychological stress. Also, the time period for the decision to have an emergency CS is usually very short due to the indication. This is often associated with minimal education of women about the subsequent procedure and the reasons for a CS by medical staff [47]. A low level of information provided to women before or during childbirth can promote a negative birth experience [41] which, in turn, may serve as a potential predictor for PTSD.

This explanation is supported by the lower proportion of women above the cut-off with Grade 3 (medically indicated) of 5.04% and Grade 4 (CS on maternal request), 0%. Both groups have a longer preparation time before the CS is performed. This is mostly accompanied by close attention and care by medical professionals during pregnancy. Consequently, there is no time constraint for decision making, nor is there a lack of education. Therefore, the subjectively perceived safety during childbirth is probably higher. However, knowledge of the need for Grade 3 medically indicated CS to avoid endangering the infant or mother, or explicit desire at Grade 4, may also strengthen acceptance and reduce mothers’ helplessness and subjective distress. Both would be predictive of PTSD, and their absence may be protective [48].

AVDs accounted for the second highest percentage (13.10%), significantly higher than vaginal births (4.27%), which may suggest that AVDs are potentially more traumatizing than previous research suggests. It can be assumed that women perceive the use of assistive devices such as delivery forceps or a suction cup as an unnatural and drastic intervention in childbirth. Also, these instruments are typically used when the birth is not progressing fast enough or when there is an imminent danger to the child. Consequently, AVD presumably leads to trauma-predictive sensations such as lack of subjective safety, helplessness, or fear for the child and is associated with an elevated PCL-5 Score [49]. This supports, for example, the research who found negative delivery experiences, inadequate education, and time pressure in the expulsion phase of women giving birth via AVD [30, 50].

The significant difference between vaginal delivery and secondary CS can probably be explained (similar to emergency CS) by the medically indicated termination of vaginal delivery, the brief decision-making window, and potential lack of education. Since neither Grade 4 nor Grade 3 showed a significant difference from the secondary CS, and they differed in particular by the urgency time criterion and preparation, could account for the slightly increased PCL-5 value of women with secondary CS.

The mean effect of the analysis of variance (ƞp² = 0.11) shows that the mode of delivery is an important factor influencing the development of a PTSD. This is also confirmed by the results of the regression analysis, in which mode of delivery alone accounted for 8.8% of the variance.

### Limitations

The study’s reliance on self-report measures limits the data’s reliability, due to the inability to verify the provided information. Other associated problems could be social desirability or the lack of clarity as to whether the questions were really understood by the participants. Furthermore, the use of PCL-5 as a PTSD measure may be a limitation, as it was not specifically designed for the postpartum period. For instance, sleep difficulties are included in the PCL-5, which might not be as appropriate or relevant in the postpartum context – where frequent disruptions to sleep are a common reality for most mothers.

One of the main strengths of this study is the great number of participants (*N* = 1223), which increases statistical power. However, almost half of the participants did not complete the study, suggesting that the study was tiring or not engaging and their responses had to be discarded. Besides limiting the number of responses available for statistical analysis, this might also create selection bias through unknown common characteristics of those who did complete the study. For instance, individuals affected by a postpartum psychiatric disorder might have a higher completion rate due to heightened personal interest, potentially introducing bias into the study results.

On the other hand, those who are experiencing mental distress while responding might avoid active confrontation. For example, a key symptom of PTSD is the avoidance of stimuli, therefore those with strong PTSD symptoms may be unlikely to take part in a study investigating their traumatic experience. Future research could potentially control this by asking participants to explain their reasons for taking part in the study. 64% of the participants were responding after the birth of their first child. This may cause a bias, as mothers are more likely to develop postpartum psychiatric disorders after the first child, than after subsequent births [51]. Thus, results may be biased and may not necessarily apply to mothers who have more than one child.

Only 1.1% of the participants had an CS on maternal request, which limits statistical power. However, this is close to the general rates in the overall German population 1.9% of the women gave birth via CS on maternal request [52], while the literature reports a rate of 2.3% [53, 54]. The CS rate of this sample is 30.6%, which is almost congruent with the CS rate in Germany in 2021 (30.9%) [55]. However, the number of AVD differed with 11.9% within the sample compared to 6.1% in the overall population [56]. Nevertheless, delivery modes should be represented equally to allow for a better and more conclusive comparison. The study categorized four CS types and two vaginal delivery types, but it did not explore other interventions during delivery that might impact the birth experience, such as amniotomy, medication during labor, etc.

Most of the participants (93.6%) were German. Therefore, the results cannot be generalized across different cultures. Consequently, replicating the study across various cultural contexts would provide a more comprehensive understanding.

The cross-sectional design does not allow for causality between higher symptom levels and the delivery mode to be examined and established. Therefore, it would be beneficial to investigate the development of postpartum mental health within a longitudinal design. Again, it is important to note that the study design means that subjects above the specified cut off values are not equated with confirmed diagnoses. The high number of participants due to the anonymous online format comes at the expense of not having the mothers physically seen by trained health professionals, leading to diagnoses that are not medically confirmed. Moreover, postpartum psychiatric disorders have been shown to decrease over time, which can only be taken into account in a longitudinal study. For example, PTSD symptoms decrease over time and only a minority of mothers fail to recover [5]. In this study, the deliveries occurred anywhere between four weeks to 12 months prior to the study. The period of time between the delivery and the study may influence the results.

Moreover, other factors unrelated to the delivery also ought to be considered. For instance, research has shown that women who experienced trauma in their past are more likely to develop PTSD [57]. Therefore, it cannot be conclusively stated that a particular mode of delivery is traumatic per se, but rather that one mode of delivery, such as emergency CS, may have a higher traumatic potential, other factors also contribute to the overall experience.

### Implications

The categorization of birth mode plays an important role in understanding the nuanced impact on maternal mental health. Our study delved into a more comprehensive classification by expanding the conventional four-category division of birth modes into a more detailed six-category framework. By subdividing the delivery modes, we gained a deeper insight into the divergent psychological implications of each mode. This nuanced approach acknowledges the distinct physiological, psychological, and emotional facets inherent the different modes, enabling a more precise analysis of their respective impacts on postpartum psychological well-being. We therefore recommend that this subdivision be used in this form in future studies. This could also help to explain previous inconsistencies in the research.

This study therefore offers a new explanation as to why the type of delivery has an impact on the development of PPD and PTSD. This has far-reaching implications. The study suggests that apart from medical requirements, the focus should be directed towards a mother’s preference, with a particular emphasis on maternal mental health, especially post-delivery.

Relative to vaginal delivery, almost every delivery mode except for CS on maternal request seems to increase the risk of PTSD. It is therefore important to carefully consider when a CS should be performed. While it can be essential and lifesaving, it should not be undertaken without thorough consideration. An increase in CS rates before holidays or the weekend due to staff shortages, convenience, or financial considerations [58] is not acceptable. Implementation should always include a risk-benefit consideration.

Several years ago, the WHO advised a permissible CS rate of 19.1% beyond which there were no demonstrated benefits in reducing maternal and neonatal mortality and morbidity compared to standard vaginal delivery [59]. With a CS rate of about 30.2% Germany is above the recommendation, which may imply that not all CS are necessary. However, the World Health Organization has undergone a significant shift in its recommendations, moving away from specific target rates. Instead, it now emphasizes addressing the individual needs of each woman during pregnancy and childbirth, suggesting non-clinical measures to reduce the unnecessary use of CS while highlighting the importance of maintaining high-quality and respectful care. These include, for example: Implementing educational interventions for active women involvement in birth planning, following evidence-based clinical guidelines with routine audits of CS practices, requiring a second medical opinion where feasible, and adopting a collaborative midwifery-obstetrician model of care [60].

It is essential to enhance the financial support for vaginal deliveries. Without such improvement, incorrect incentives may be established, potentially compromising the ability to make an unbiased decision regarding a potential CS. Also, this decision should not be made solely on the basis of staff shortage or time pressure on the part of the hospital team but should always be based on medical necessity and especially on the preferences of the woman, since the subjective birth experience has the greatest influence on the development of PTSD after delivery [19].

Routine screening for PPD and PTSD symptoms in mothers in the first postpartum year may lead to earlier intervention and possibly prevent further mental health impairment. As secondary CS were shown to increase the risk of PPD, one could argue that after a secondary CS mothers should also be checked for symptoms of depression. However, as there is no research yet to explain why different types of CS have different effects, routine screening should be performed in general. Future research could investigate which aspects of the different delivery modes impact maternal mental health and explore how the perception of birth may be influenced by a specific delivery mode.

## Data Availability

The datasets analyzed during the current study are not publicly available because longitudinal data was collected within the framework of the project, which is currently still being evaluated, but are available from the corresponding author on reasonable request.

## References

[1] Dorn A, Mautner C (2018). Postpartale Depress Der Gynäkologe.

[2] Howard LM, Molyneaux E, Dennis CL, Rochat T, Stein A, Milgrom J (2014). Non-psychotic mental disorders in the perinatal period. Lancet (London England).

[3] Shorey S, Chee CYI, Ng ED, Chan YH, Tam S, Chong YS (2018). Prevalence and incidence of postpartum depression among healthy mothers: a systematic review and meta-analysis. J Psychiatr Res.

[4] Chen J, Cross WM, Plummer V, Lam L, Tang S (2019). A systematic review of prevalence and risk factors of postpartum depression in Chinese immigrant women. Women Birth.

[5] Dekel S, Stuebe C, Dishy G (2017). Childbirth induced posttraumatic stress syndrome: a systematic review of prevalence and risk factors. Front Psychol.

[6] Yildiz PD, Ayers S, Phillips L (2017). The prevalence of posttraumatic stress disorder in pregnancy and after birth: a systematic review and meta-analysis. J Affect Disord.

[7] Johansson M, Benderix Y, Svensson I (2020). Mothers’ and fathers’ lived experiences of postpartum depression and parental stress after childbirth: a qualitative study. Int J Qualitative Stud Health well-being.

[8] Bullinger-Naber M, Naber D. (1999). Erfassung der Lebensqualität psychisch Kranker. In Helmchen, H. Herausgeber (1999). Psychiatrie der Gegenwart. Band 2. (S. 235–260). Berlin: Springer.

[9] Nicholls K, Ayers S (2007). Childbirth-related post-traumatic stress disorder in couples: a qualitative study. Br J Health Psychol.

[10] Onoye JM, Shafer LA, Goebert DA, Morland LA, Matsu CR, Hamagami F (2013). Changes in PTSD symptomatology and mental health during pregnancy and postpartum. Archives of Women’s Mental Health.

[11] Bhatia MS (2020). Maternal risk markers of postnatal depression. J Postgrad Med.

[12] Furtado M, Van Lieshout RJ, Van Ameringen M et al. (2019). Biological and psychosocial predictors of anxiety worsening in the postpartum period: a longitudinal study. J Affect Disord. 2019;250:218–225. 10.1016/j.jad.2019.02.064 10.1016/j.jad.2019.02.064 30870771

[13] Goodwin AI, Welchman CL, Mead J, Hines KN, Quinn KH (2019). Risk factors for the development of Antepartum & Postpartum Depression in patients hospitalized during pregnancy [16D]. Obstet Gynecol.

[14] Hernández-Martínez A, Rodríguez-Almagro J, Molina-Alarcón M, Infante-Torres N, Manzanares MD, Martínez-Galiano JM (2019). Postpartum post-traumatic stress disorder: Associated perinatal factors and quality of life. J Affect Disord.

[15] Ghaedrahmati M, Kazemi A, Kheirabadi G, Ebrahimi A, Bahrami M (2017). Postpartum depression risk factors: a narrative review. J Edu Health Promot.

[16] Grekin R, O’Hara MW (2014). Prevalence and risk factors of postpartum posttraumatic stress disorder: a meta-analysis. Clin Psychol Rev.

[17] Howard LM, Molyneaux E, Dennis C-L, Rochat T, Stein A, Milgrom J (2014). Non-psychotic mental disorders in the perinatal period. The Lancet.

[18] Ayers S, Bond R, Bertullies S, Wijma K (2016). The aetiology of post-traumatic stress following childbirth: a meta-analysis and theoretical framework. Psychol Med.

[19] Garthus-Niegel S, von Soest T, Vollrath ME, Eberhard-Gran M (2013). The impact of subjective birth experiences on post-traumatic stress symptoms: a longitudinal study. Arch Women Ment Health.

[20] Beck-Hiestermann FML, Gries S, Mehl S et al. (2023). Adverse childbirth experiences - results of an online survey of woman during their first year postpartum, 04 October 2023, PREPRINT (Version 1) available at Research Square [10.21203/rs.3.rs-3408649/v1]

[21] Lobel M, DeLuca RS. Psychosocial sequelae of cesarean delivery: review and analysis of their causes and implications. Social science & medicine (1982), 2007;64(11):2272–2284. 10.1016/j.socscimed.2007.02.028 10.1016/j.socscimed.2007.02.028 17395349

[22] Xu H, Ding Y, Ma Y, Xin X, Zhang D (2017). Cesarean section and risk of postpartum depression: a meta-analysis. J Psychosom Res.

[23] Baghianimoghadam MH, Shodjaee zadeh D, Aminian AH (2009). Caesarean section, vaginal delivery and Post Natal Depression. Iran J Publ Health.

[24] Yang XJ, Sun SS (2017). Comparison of maternal and fetal complications in elective and emergency cesarean section: a systematic review and meta-analysis. Arch Gynecol Obstet.

[25] Swain JE, Tasgin E, Mayes LC, Feldman R, Constable RT, Leckman JF (2008). Maternal brain response to own baby-cry is affected by cesarean section delivery. J Child Psychol Psychiatry Allied Discip.

[26] Schmidt D, Seehagen S, Hirschfeld G (2017). Repetitive negative thinking and impaired mother–infant bonding: a longitudinal study. Cogn Ther Res.

[27] Carter FA, Frampton CM, Mulder RT (2006). Cesarean section and postpartum depression: a review of the evidence examining the link. Psychosom Med.

[28] Goker A, Yanikkerem E, Demet MM, Dikayak S, Yildirim Y, Koyuncu FM (2012). Postpartum Depression: is Mode of Delivery a risk factor?. ISRN Obstet Gynecol.

[29] Faisal-Cury A, Menezes PR (2019). Type of delivery is not associated with maternal depression. Arch Women Ment Health.

[30] De Klerk HW, Boere E, van Lunsen RH, Bakker JJH (2017). Women’s experiences with vaginal examinations during labor in the Netherlands. J Psychosom Obstet Gynecol.

[31] Gamble J, Creedy DK (2005). Psychological trauma symptoms of operative birth. Br J Midwifery.

[32] RCOG. (2010) Good practice 11 classification of urgency of CS – a continuum of risk. London. RCOG Press, in: NICE-guideline [NG192], Caesarean birth, Published: 31 March 2021 Last updated: 06 September 2023, retrieved from: https://www.nice.org.uk/guidance/ng192

[33] Cox JL, Holden JM, Sagovsky R (1987). Detection of postnatal depression. Development of the 10-item Edinburgh postnatal depression scale. Br J Psychiatry.

[34] Bergant AM, Nguyen T, Heim K, Ulmer H, Dapunt O (1998). Deutschsprachige Fassung und Validierung Der Edinburgh postnatal depression scale. Dtsch Med Wochenschr.

[35] Weathers FW, Litz BT, Keane TM, Palmieri PA, Marx BP, Schnurr PP. (2013). The PTSD Checklist for *DSM-5* (PCL-5). Scale available from the National Center for PTSD at www.ptsd.va.gov.

[36] Krüger-Gottschalk A, Knaevelsrud C, Rau H (2017). The German version of the posttraumatic stress disorder checklist for DSM-5 (PCL-5): psychometric properties and diagnostic utility. BMC Psychiatry.

[37] Cohen J (1988). Statistical Power Analysis for the behavioral sciences.

[38] O’Hara MW, McCabe JE (2013). Postpartum depression: current status and future directions. Ann Rev Clin Psychol.

[39] Schneider DA (2018). Birthing failures: Childbirth as a female Fault line. J Perinat Educ.

[40] Hüner B, Schmiedhofer M, Derksen C, Polasik A, Janni W, Reister F, Lippke S (2023). „Helplessness, giving up of any self-responsibility and self-determination - a qualitative evaluation of Traumatizing Birth experiences in Relation to Birth Mode]. Z für Geburtshilfe Und Neonatologie.

[41] Handelzalts JE, Peyser W, Krissi A, Levy H, Wiznitzer S, Peled Y (2017). Indications for emergency intervention, Mode of Delivery, and the Childbirth experience. PLoS ONE.

[42] Ayers S, Jessop D, Pike A, Parfitt Y, Ford E (2014). The role of adult attachment style, birth intervention and support in posttraumatic stress after childbirth: a prospective study. J Affect Disord.

[43] Huber G, Seelbach-Göbel B (2009). Die postpartale depression – ist ein Screening Durch die Geburtsklinik Sinnvoll?. Geburtshilfe Frauenheilkd.

[44] Martini J, Asselmann E, Einsle F, Strehle J, Wittchen HU (2016). A prospective-longitudinal study on the association of anxiety disorders prior to pregnancy and pregnancy- and child-related fears. J Anxiety Disord.

[45] Kühner C (2016). Psychiatrische Erkrankungen in Schwangerschaft Und Stillzeit. Nervenarzt.

[46] King L, McKenzie-McHarg K, Horsch A (2017). Testing a cognitive model to predict posttraumatic stress disorder following childbirth. BMC Pregnancy Childbirth.

[47] Berg D. Organisation der Geburtshilflichen Versorgung. Gynäkologe. 2013;46(80). 10.1007/s00129-012-2998-5

[48] Di Blasio P, Miragoli S, Camisasca E, Di Vita AM, Pizzo R, Pipitone L (2015). Emotional distress following childbirth: an intervention to buffer depressive and PTSD symptoms. Europe’s J Psychol.

[49] Muraca GM, Ralph LE, Christensen P, D’Souza R, Geoffrion R, Lisonkova S, Joseph KS (2023). Maternal and neonatal trauma during forceps and vacuum delivery must not be overlooked. BMJ.

[50] Wiklund I, Edman G, Ryding EL, Andolf E (2008). Expectation and experiences of childbirth in primiparae with caesarean section. BJOG: An International Journal of Obstetrics and Gynaecology.

[51] Nakano M, Upadhyaya S, Chudal R (2019). Risk factors for impaired maternal bonding when infants are 3 months old: a longitudinal population based study from Japan. BMC Psychiatry.

[52] Bertelsmann Foundation. Health fact check. Caesarean births – development and regional distribution. 2015. Retrieved from: https://faktencheck-gesundheit.de/fileadmin/files/BSt/Publikationen/GrauePublikationen/ GP_Faktencheck_Gesundheit_Kaiserschnitt.pdf

[53] Robson SJ, Tan WS, Adeyemi A, Dear KB (2009). Estimating the rate of cesarean section by maternal request: anonymous survey of obstetricians in Australia. Birth (Berkeley Calif).

[54] Turner CE, Young JM, Solomon MJ, Ludlow J, Benness C, Phipps H (2008). Vaginal delivery compared with elective caesarean section: the views of pregnant women and clinicians. BJOG: An International Journal of Obstetrics and Gynaecology.

[55] German Federal Statistics Office. Press Release No. N 009 of February 15, 2023: Nearly one-third of all births in 2021 by cesarean section. 2021. Retrieved from https://www.destatis.de/DE/Themen/Gesellschaft-Umwelt/Gesundheit/Krankenhaeuser/Tabellen/krankenhausentbindungen-kaiserschnitt.html

[56] Statista. Number of births by vacuum extraction (suction cup) in Germany from 2004 to 2021. 2021. Retrieved from https://de.statista.com/statistik/daten/studie/448735/umfrage/anzahl-der-geburten-durch-vakuumextraktion-in-deutschland/

[57] Orovou E, Dagla M, Iatrakis G, Lykeridou A, Tzavara C, Antoniou E (2020). Correlation between Kind of Cesarean Section and posttraumatic stress disorder in Greek Women. Int J Environ Res Public Health.

[58] Halla M, Mayr H, Pruckner GJ, García-Gómez P (2020). Cutting fertility? Effects of cesarean deliveries on subsequent fertility and maternal labor supply. J Health Econ.

[59] Molina G, Weiser TG, Lipsitz SR, Esquivel MM, Uribe-Leitz T, Azad T, Shah N, Semrau K, Berry WR, Gawande AA, Haynes AB (2015). Relationship between cesarean delivery rate and maternal and neonatal mortality. JAMA.

[60] WHO Recommendations Departmental News: Caesarean section rates continue to rise, amid growing inequalities in access - Rising rates suggest increasing numbers of medically unnecessary, potentially harmful procedures. Geneva: World Health Organization; 2021. Available from: https://www.who.int/news/item/16-06-2021-caesarean-section-rates-continue-to-rise-amid-growing-inequalities-in-access

